# Influence of 2-hydroxyethylammonium acetate-based protic ionic liquids on the thermophysical properties of aqueous *DL-*alanine solutions

**DOI:** 10.1186/s13065-025-01603-1

**Published:** 2025-08-13

**Authors:** Mohammad Amin Morsali, Hemayat Shekaari

**Affiliations:** Department of Physical Chemistry, Faculty of Chemistry, University of Tabriz, Tabriz, 5166616471 Iran

**Keywords:** *DL-alanine*, Protic ionic liquids, Volumetric properties, Viscosity, Molar refractionon, COSMO Analysis

## Abstract

Understanding the hydration behavior of amino acids is fundamental to gaining insights into protein solvation mechanisms. Within this framework, examining the solvation properties of amino acids in aqueous media containing protic ionic liquids (PILs) a novel class of environmentally friendly solvents is crucial for characterizing their hydration dynamics. This research focuses on the influence of ammonium-based PILs, specifically mono-, bis-, and tris-(2-hydroxyethyl) ammonium acetate, on the hydration characteristics of *DL-*alanine. By employing COSMO computational analysis alongside thermophysical property measurements, the study evaluates the hydration energies and σ-profiles of *DL-*alanine and the PILs. The thermophysical behavior of *DL-*alanine in aqueous solutions containing varying concentrations of protic ionic liquids (PILs) was systematically analyzed using experimental data and modeling approaches. The density, speed of sound, viscosity, and refractive index were measured for ternary solutions of *DL-*alanine, water, and PILs across a temperature range of (298.15 to 318.15) K under atmospheric pressure. The standard partial molar volume ($\:{{V}_{\phi\:}}_{}^{0}$) measured over the studied temperature range indicates that the solute-solvent interactions between [2-HEA]Ac and *DL-*alanine in the presence of water is 63.119 cm^3^·mol^-1^. Additionally, the hydration layer surrounding *DL-*alanine is notably influenced by temperature, as higher temperatures lead to the release of more water molecules relative to PIL-containing solutions. This temperature-dependent effect is especially pronounced in the presence of (2-hydroxyethyl) ammonium acetate, underscoring its significant impact on the hydration behavior of *DL-*alanine.

**Supplementary Information:**

The online version contains supplementary material available at 10.1186/s13065-025-01603-1.

## Introduction

Water is fundamental to the structure, stability, dynamics, and functionality of biological macromolecules, including proteins and DNA [1]. Water plays an active role in protein folding, molecular recognition, and the assembly of macromolecular structures. The hydrogen bonds involved in these processes, while individually weak, exert significant collective strength, rendering them particularly well-suited for facilitating molecular recognition and structural organization within biological systems [2, 3]. Water functions not merely as a passive solvent but as an integral component of biomolecular systems, influencing their structures while simultaneously being influenced by them [4]. A comprehensive understanding of water’s role is essential for advancing the design and functional understanding of proteins and nucleic acids. The amphoteric nature of amino acids and their hydration characteristics have been extensively analyzed using various experimental techniques [5, 6]. Evidence from NMR spectroscopy and crystallographic studies indicates that amino acids in their cationic form exhibit greater hydration compared to their zwitterion form, with distinct hydrogen bonding patterns identified in crystal structures. Additionally, the hydropathic properties of amino acid side chains in protein environments have been elucidated through investigations of structural changes in water networks, offering valuable insights into local hydrophobicity and hydrophilicity [7, 8].

Protic ionic liquids (PILs) have gained recognition as versatile designer solvents, distinguished by their unique properties derived from extensive hydrogen bonding networks. These networks significantly influence the physicochemical properties of PILs, including density, thermal stability, viscosity, and conductivity [9, 10]. The incorporation of water into PILs further enhances their solvent characteristics, notably by reducing viscosity and increasing conductivity. As research progresses, PILs are anticipated to play a pivotal role in advancing sustainable chemical processes and renewable energy technologies [11]. Protic ionic liquids (PILs) and their interactions with water have attracted considerable interest owing to their distinctive properties and diverse applications. The selection of mono-, bis-, and tris-substituted forms of 2-hydroxyethylammonium acetate allows us to investigate the influence of substitution on solvation properties and hydrogen bonding capabilities. This variation enables a comprehensive analysis of how molecular structure affects the physicochemical behavior of protic ionic liquids. Understanding these differences can provide insights into the design of more effective solvent systems for amino acid interactions [12, 13].

Understanding the molecular interactions between protic ionic liquids (PILs) and biomolecules is crucial for designing effective PIL-based processes. *DL*-alanine is a non-polar, aliphatic amino acid that plays a crucial role in protein synthesis and serves as a building block for peptides. It exists in two enantiomeric forms, D- and L-alanine, both of which are involved in various metabolic pathways. Due to its small size and simple structure, *DL*-alanine is often used in studies of solvation dynamics and interactions with solvents. Its unique properties make it a valuable model compound for investigating the effects of different solvent systems, including protic ionic liquids. Although significant progress has been made in exploring protein-PIL interactions, research on peptide-PIL interactions is still in its early stages. Both experimental and computational approaches are being utilized to uncover the underlying mechanisms [14]. Given the complexity of proteins composed of numerous amino acids studying individual amino acids offers a simpler and more focused approach than examining whole proteins. Investigating the thermophysical properties of amino acids in systems containing protic ionic liquids and water provides valuable insights into solute-solvent and solute-solute interactions. In recent years, significant research has focused on the thermodynamic behavior of mixtures of amino acids and first-generation ionic liquids in aqueous media across various temperature ranges [15–17]. The susceptibility of amino acids to denaturation has driven studies on their thermodynamic properties in water, particularly in the presence of protic ionic liquids, highlighting the protective effects these unique solvents can exert against amino acid denaturation [18, 19]. The study of interactions between *DL*-alanine in polar solvents such as water and protic ionic liquids is crucial for understanding solvation phenomena. Water, as a polar solvent, facilitates significant hydrogen bonding interactions with amino compounds like *DL*-alanine, enhancing solubility and stability. Protic ionic liquids, due to their ionic nature and ability to form hydrogen bonds, further contribute to these interactions. Factors such as temperature and solvent concentration can influence the equilibrium of these interactions, thereby affecting the physical and chemical properties of the system. The chemical structure of *DL*-alanine, including its various functional groups, also plays a vital role in determining the nature and strength of these interactions. Spectroscopic techniques, such as NMR and FTIR, provide valuable insights into the interactions and structural changes occurring within the system. Understanding these interactions has practical implications in industrial processes and pharmaceuticals, while computational modeling can offer predictive insights into the behavior of these systems. The specific interactions facilitated by PILs, such as enhanced hydrogen bonding and altered solvation dynamics, can significantly influence amino acid stability and reactivity. By elucidating these effects, we can better understand how PILs modify the properties of amino acids compared to traditional solvents. This insight is crucial for advancing the design of solvent systems in biochemical applications [20, 21].

This study explores the interactions between *DL-*alanine and specific protic ionic liquids (PILs) namely, 2-hydroxyethylammonium acetate, bis (2-hydroxyethylammonium acetate), and tris (2-hydroxyethylammonium acetate) in aqueous solutions. Experimental measurements were carried out to determine the density, speed of sound, viscosity, and refractive index of aqueous *DL-*alanine solutions with varying concentrations of these PILs. The experiments were conducted over a temperature range of (288.15 K to 318.15) K under atmospheric pressure. From the experimental data, key thermodynamic and physical parameters, including the partial molar volume ($\:{v}_{\phi\:}^{0}$), partial molar isentropic compressibility ($\:{\kappa\:}_{\phi\:}^{0}$), viscosity *B*-coefficient (*B*), and molar refraction (*R*_*M*_*)*, were calculated. Additionally, COSMO computational methods were employed to elucidate the bonding interactions and intrinsic properties of the species, analyzed through σ-profiles and related theoretical results.

## Experimental

### Materials

All reagents utilized in this study, including *DL-*alanine, monoethanolamine, diethanolamine, triethanolamine, and acetic acid, were procured from Merck and used as received, without additional purification. Deionized ultrapure water, with a specific conductance of less than 1 µS·cm^− 1^, was employed for the preparation of aqueous solutions containing the amino acid and ionic liquids. Detailed information on the reagents and their specifications is provided in Table 1:

**Table 1:**
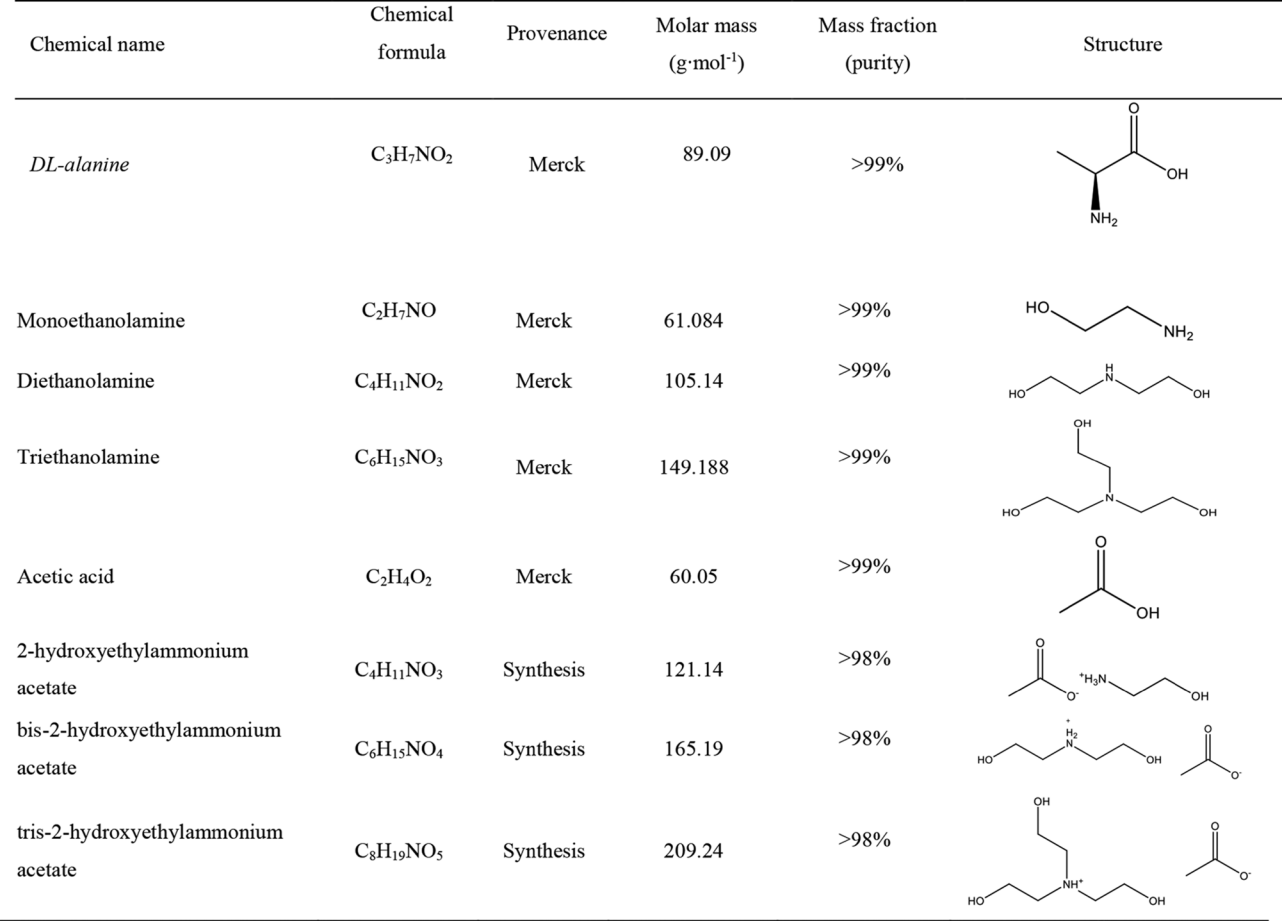
Descriptions of the used chemicals

### PILs synthesis and purification

In the present study, the synthesis and purification of ionic liquids, specifically 2-hydroxyethylammonium acetate, bis (2-hydroxyethyl) ammonium acetate, and tris (2-hydroxyethyl) ammonium acetate, were conducted. The synthesis process involved the controlled, dropwise equimolar (1:1 molar ) addition of acetic acid to ethanolamine, which was maintained in an ice bath. The reaction mixture was vigorously stirred at room temperature for 24 h using a magnetic stirrer. To ensure high purity, the synthesized ionic liquids were subjected to vacuum drying for 3 h using a D25 vacuum pump (USA). The resulting protic ionic liquids (PILs) were rigorously characterized by proton nuclear magnetic resonance (^1^H NMR) and Fourier-transform infrared (FT-IR) spectroscopy. Additionally, their water content was precisely determined through Karl-Fischer titration. Detailed characterization results are provided in the supporting information section. As stated thorough the caractrization method (FT-IR, H-NMR, KARL FISHER) the synthesized PILs in this study presented high quality of 98% purity and affirmational synthesized rate gave us 93% yield.

### Apparatus and procedure

The solutions were prepared with meticulous precision using a Shimadzu AW-220 analytical balance, ensuring a measurement precision of ± 2 × 10^− 4^ g. Density and speed of sound were measured with a digital vibrating U-shaped densitometer (Anton Paar DSA5000), providing resolutions of 1 × 10^− 6^ g·cm^− 3^ for density and 0.01 m·s^− 1^ for speed of sound. The associated measurement uncertainties were 4 × 10^− 5^ g·cm^− 3^ for density and ± 0.7 m·s^− 1^ for speed of sound. The reported uncertainty has been calculated thorough the instructions of the national institute of technology (NIST). The apparatus features an integrated thermostat based on Peltier technology, ensuring precise temperature control within ± 0.05 K. The instrument was calibrated using air and distilled water, and speed of sound measurements were conducted at a frequency of 3 MHz.

Viscosity was measured with a digital microviscometer, calibrated at 298.15 K using doubly distilled water. The apparatus features an integrated thermostat based on Peltier technology, ensuring precise temperature control within ± 0.05 K. The refractive index was determined using a digital refractometer (Mettler Toledo) with an accuracy of ± 0.0002 units; calibration was performed twice with doubly distilled water.

For computational analysis, density functional theory (DFT) calculations were performed using the Dmol³ module. The geometries of the protic ionic liquids (PILs) were optimized using the generalized gradient approximation Vosko-Wilk-Nusair-Becke-Perdew (GGA VWN-BP) functional, which combines the BP functional with the VWN framework for local correlation. The COSMO method was employed in a two-step process involving initial geometry optimization followed by energy optimization.

## Results and discussion

### Molecular fingerprint: σ-Profile

The sigma profile (σ-profile) is a critical concept in COSMO (Conductor-like Screening Model) calculations, widely used to characterize molecular interactions in various chemical environments [22, 23]. In COSMO calculations, the sigma profile represents the distribution of surface charge densities on the molecular surface of a solute. This distribution is derived from quantum chemical simulations, where the molecule is placed in a virtual conductor medium. The conductor environment ensures that the molecular surface is fully polarized, facilitating accurate predictions of interaction energies in non-conducting solvents [24]. The sigma profile is constructed by dividing the molecular surface into segments and calculating the local charge on each segment. The results are then expressed as a histogram, where the sigma-values are plotted against their respective surface area fractions. This histogram provides a quantitative representation of the molecule’s polarity and its capacity to engage in hydrogen bonding, van der Waals interactions, and electrostatic forces.

**Fig. 1:**
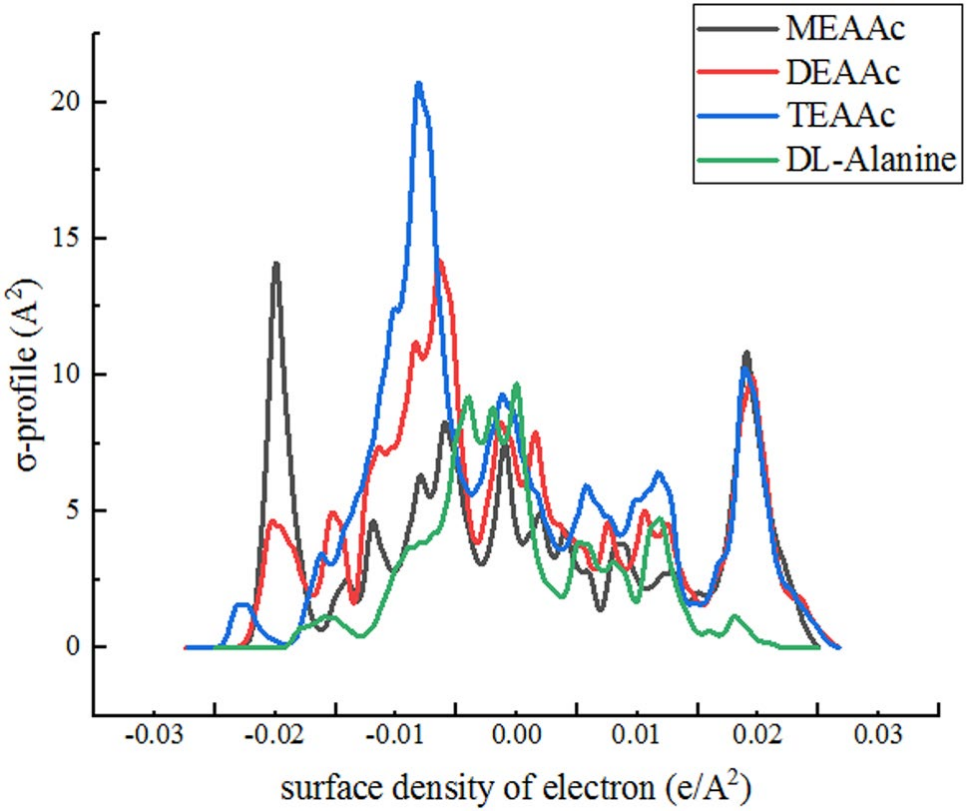
Sigma profiles of *DL-alanine*, water, [2-HEA] Ac, [BHEA] Ac, and [THEA] Ac

The GGA VWN-BP functional, implemented within the Dmol³ framework as recommended by its developers, has demonstrated effectiveness in modeling realistic solvent systems [25]. In this study, COSMO calculations were conducted using density functional theory (DFT) within the Dmol³ module of Materials Studio (Biovia, Materials Studio 2023). Molecular geometry optimization was performed using the GGA VWN-BP functional to ensure accurate structural representations. The σ-profiles for the investigated solvents and protic ionic liquids (PILs), are presented in Fig. 1:

The calculated cavity volume, cavity surface area, and HOMO and LUMO energy levels of the compounds are summarized in Table 2:

**Table 2:** The cavity volume and surface beside hydration energy, and highest occupied molecular orbital numbers and energy, lowest unoccupied molecular orbital of the studied compounds

Compound	Cavity volume	Cavity surface	Dielectric (hydration) energy	*n*_HOMO_	HOMO	*n*_LUMO_	LUMO
	Å^3^	Å^2^	kcal/mol		eV		eV
*DL* *-alanine*	105.1877	123.0834	-12.76	24	-5.725eV	25	-1.130 eV
[2-HEA]Ac	155.2485	188.5375	-114.85	33	-4.457 eV	34	0.498 eV
[BHEA]Ac	207.5569	241.6801	-111.79	45	-4.439 eV	46	0.226 eV
[THEA]Ac	251.1792	271.4756	-103.36	57	-4.444 eV	58	-0.047 eV

The Generalized Gradient Approximation (GGA) VWN-BP functional, which integrates the Vosko-Wilk-Nusair (VWN) correlation with the Becke-Perdew (BP) exchange, exhibits several limitations that may affect its applicability in computational studies. Notably, it struggles with systems characterized by strong electron correlation, such as transition metal oxides, often resulting in underestimations of binding energies and failing to capture essential physical phenomena. Additionally, the functional inadequately addresses long-range dispersion interactions, which are critical in systems with weak van der Waals forces, leading to inaccuracies in molecular geometries and interaction energies. Furthermore, the presence of self-interaction errors can compromise predictions for systems with unoccupied states, while the treatment of exchange-correlation effects remains limited, particularly in non-uniform electron density distributions. Within the Dmol³ framework, the functional is employed to optimize molecular geometries, calculate electronic properties, and perform DFT-based analyses. Its reliability and precision make it particularly effective for studying solvation behaviors in realistic solvent environments [26].

In this study, water was used as the solvent for all calculations, providing critical insights into the solvation and interaction properties of the systems under investigation. *DL-*alanine exhibited the smallest cavity volume and surface area among the analyzed molecules, consistent with its compact molecular structure. This compactness was also reflected in its relatively less negative dielectric (hydration) energy, indicating weaker interactions with the solvent compared to the other studied compounds.

In contrast, the mono-, bis-, and tris-(2-hydroxyethyl) ammonium acetate compounds displayed progressively larger cavity volumes and surface areas, reflecting their greater spatial demands and more complex interactions with the solvent. These compounds also exhibited increasingly negative dielectric (hydration) energies, suggesting stronger and more favorable interactions with the solvent environment. Furthermore, all the molecules demonstrated negative HOMO energy levels, while significant variations in their LUMO energy levels were observed. These differences highlight variations in their electronic properties and potential reactivity.

### Volumetric properties

The densities of ternary aqueous solutions containing *DL-*alanine and mono-, bis-, and tris-(2-hydroxyethyl)ammonium acetate were measured at atmospheric pressure over a temperature range of (288.15 to 318.15) K, as shown in Table 3:

**Table 3:** The density of ternary solutions containing *DL-*alanine in aqueous PILs and apparent molar volume of *DL-*alanine in various concentration of PILs and temperature range of (288.15 to 318.15) K under atmospheric pressure.

*m*	d / kg·m^− 3^				10^6^ V_φ_ / m^3·^mol^− 1^			
	288.15 K	298.15 K	308.15 K	318.15 K	288.15 K	298.15 K	308.15 K	318.15 K
[2-HEA]Ac 0.0501 (mol·kg^-1^)								
0.0000	1000.871	998.185	995.618	993.289				
0.0495	1002.276	999.556	996.970	994.627	60.596	61.373	61.847	62.215
0.0999	1003.712	1000.947	998.343	995.990	60.451	61.333	61.794	62.118
0.1493	1005.120	1002.313	999.694	997.327	60.358	61.260	61.699	62.038
0.1994	1006.551	1003.689	1001.052	998.678	60.240	61.217	61.659	61.969
0.2492	1007.976	1005.065	1002.401	1000.017	60.122	61.120	61.602	61.907
0.2993	1009.397	1006.446	1003.736	1001.374	60.065	61.047	61.618	61.811
[2-HEA]Ac 0.1001 (mol·kg^-1^)								
0.0000	1002.144	999.434	996.854	994.539				
0.0499	1003.576	1000.826	998.227	995.886	60.261	61.149	61.619	62.225
0.0996	1004.986	1002.199	999.592	997.232	60.323	61.185	61.545	62.081
0.1497	1006.408	1003.581	1000.964	998.579	60.286	61.158	61.494	62.047
0.1995	1007.806	1004.943	1002.326	999.925	60.294	61.153	61.427	61.944
0.2494	1009.179	1006.296	1003.649	1001.274	60.390	61.177	61.536	61.860
0.3005	1010.587	1007.683	1005.050	1002.653	60.412	61.152	61.418	61.775
[2-HEA]Ac 0.1501 (mol·kg^-1^)								
0.0000	1003.427	1000.690	998.098	995.779				
0.0496	1004.799	1002.038	999.420	997.071	61.233	61.809	62.425	63.119
0.1001	1006.202	1003.416	1000.769	998.401	61.063	61.645	62.288	62.866
0.1499	1007.602	1004.783	1002.113	999.752	60.865	61.505	62.118	62.484
0.1999	1009.010	1006.173	1003.481	1001.093	60.706	61.299	61.892	62.323
0.2493	1010.382	1007.536	1004.834	1002.443	60.660	61.189	61.723	62.097
0.2988	1011.791	1008.924	1006.174	1003.801	60.480	61.007	61.629	61.893
[BHEA]Ac 0.0498 (mol·kg^-1^)								
0.0000	1001.252	998.555	995.977	993.657				
0.0500	1002.686	999.953	997.359	995.034	60.115	60.927	61.337	61.516
0.1004	1004.131	1001.357	998.751	996.419	60.121	60.979	61.347	61.546
0.1494	1005.561	1002.748	1000.132	997.792	60.117	60.980	61.321	61.534
0.1998	1006.943	1004.092	1001.468	999.124	60.165	61.029	61.349	61.549
0.2489	1008.372	1005.469	1002.841	1000.476	60.133	61.051	61.340	61.602
0.3000	1009.754	1006.817	1004.165	1001.798	60.150	61.047	61.384	61.623
[BHEA]Ac 0.0999 (mol·kg^-1^)								
0.0000	1002.993	1000.256	997.647	995.313				
0.0504	1004.445	1001.681	999.043	996.690	60.102	60.724	61.387	61.847
0.0999	1005.857	1003.071	1000.402	998.037	60.144	60.723	61.413	61.806
0.1495	1007.264	1004.454	1001.761	999.383	60.177	60.754	61.406	61.783
0.1993	1008.665	1005.823	1003.117	1000.729	60.194	60.810	61.388	61.742
0.2496	1010.075	1007.205	1004.479	1002.082	60.196	60.819	61.380	61.716
0.2994	1011.449	1008.545	1005.812	1003.418	60.243	60.893	61.399	61.682
[BHEA]Ac 0.1498 (mol·kg^-1^)								
0.0000	1004.617	1001.840	999.210	996.863				
0.0499	1006.042	1003.228	1000.589	998.225	60.316	61.144	61.413	61.835
0.1007	1007.476	1004.631	1001.982	999.597	60.375	61.139	61.417	61.877
0.1501	1008.869	1005.985	1003.329	1000.929	60.369	61.172	61.434	61.871
0.2004	1010.265	1007.352	1004.691	1002.279	60.415	61.185	61.429	61.837
0.2497	1011.647	1008.691	1006.012	1003.601	60.364	61.174	61.460	61.801
0.2995	1013.015	1010.032	1007.325	1004.912	60.393	61.175	61.523	61.827
[THEA]Ac 0.0501 (mol·kg^-1^)								
0.0000	1002.423	999.709	997.119	994.789				
0.0497	1003.861	1001.123	998.489	996.119	60.008	60.576	61.551	62.444
0.0979	1005.239	1002.465	999.801	997.385	60.072	60.772	61.620	62.590
0.1498	1006.701	1003.890	1001.199	998.737	60.209	60.945	61.712	62.687
0.1994	1008.060	1005.226	1002.522	999.998	60.412	61.105	61.770	62.840
0.2501	1009.435	1006.562	1003.838	1001.285	60.553	61.282	61.913	62.903
0.3003	1010.822	1007.891	1005.094	1002.528	60.532	61.351	62.139	63.024
[THEA]Ac 0.0998 (mol·kg^-1^)								
0.0000	1005.271	1002.492	999.862	997.507				
0.0499	1006.682	1003.878	1001.224	998.844	60.578	61.167	61.736	62.321
0.0996	1008.082	1005.250	1002.576	1000.177	60.543	61.163	61.695	62.221
0.1501	1009.486	1006.632	1003.940	1001.536	60.568	61.158	61.662	62.073
0.1993	1010.845	1007.983	1005.276	1002.861	60.620	61.128	61.605	61.990
0.2492	1012.213	1009.332	1006.599	1004.189	60.641	61.142	61.647	61.952
0.2994	1013.559	1010.687	1007.972	1005.554	60.734	61.137	61.512	61.806
[THEA]Ac 0.1502 (mol·kg^-1^)								
0.0000	1008.077	1005.240	1002.572	1000.192				
0.0499	1009.452	1006.591	1003.909	1001.526	61.207	61.779	62.151	62.296
0.0999	1010.831	1007.949	1005.249	1002.864	61.077	61.620	62.032	62.166
0.1499	1012.233	1009.315	1006.593	1004.201	60.854	61.485	61.937	62.101
0.1948	1013.474	1010.546	1007.801	1005.399	60.789	61.349	61.835	62.032
0.2498	1015.006	1012.072	1009.310	1006.887	60.671	61.152	61.619	61.875
0.2997	1016.448	1013.450	1010.661	1008.224	60.393	61.022	61.517	61.790

The standard uncertainties for molality, temperature and pressure were *u* (*m*) *=* 0.001 mol·kg^− 1^, *u* (*T*) *=* 0.2 K, *u* (*P*) *=* 10.5 hPa, respectively with level of confidence 0.68. The standard combined uncertainty for density and apparent molar volume were about, *u* (*d*) *=* 0.06 × 10^− 4^ kg·m^− 3^ and *u* (*V*_*φ*_) = 5 × 10^− 6^ m^3^·mol^− 1^ (level of confidence 0.68), respectively

**Fig. 2:**
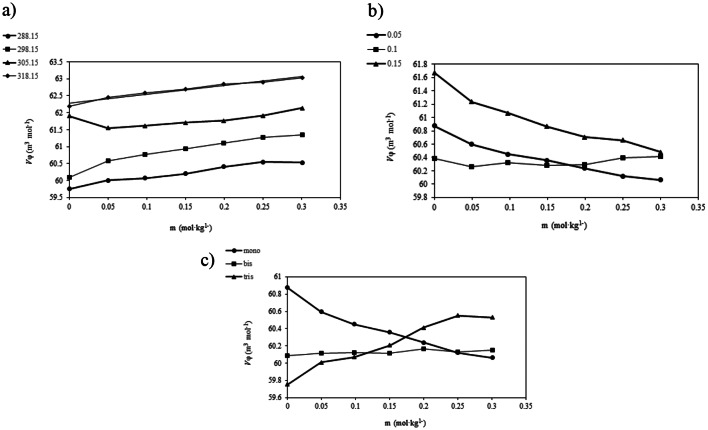
Variation of apparent molar volumes( $V\phi$ )with, **a**) temperature, **b**) concentration of PILs and **c**) cation size of the PILs

The apparent molar volumes of the studied solutions were calculated using the Eq. (1) provided in reference [27], with the resulting values presented in Table 3:

1 $$\:{V}_{\phi\:}=\frac{M}{d}-\left[\frac{\left(d-{d}_{0}\right)}{md{d}_{0}}\right]$$

where, Molar mass of amino acids is shown by *M* (kg·mol^-1^), and density of solution and density of solvents (water + PILs) are represented by *d* and *d*_0​_ (kg·m^-3^), respectively. Also *m* is the molality of the experimental solution (mol·kg^-1^). The thermodynamic properties of the investigated solutions were analyzed to provide a comprehensive understanding of their behavior. An example depicting the variation of ($\:{V}_{\phi\:}$) ​ with the molality of *DL-*alanine in the coexistence of the PILs, their concentrations, and temperature is presented in Fig. 2:

 Masson model has been used to calculate the apparent molar properties, as expressed by the Eq. (2):

2 $$\:{V}_{\phi\:}={V}_{\phi\:}^{0}+{S}_{v}m$$

The parameters ($\:{V}_{\phi\:}^{0}$), ($\:{S}_{v}$) presented in Table 4, correspond to the ternary aqueous solutions of PILs. The term ($\:{V}_{\phi\:}^{0}$)​represents the intercept of Eq. (2), referred to as the standard partial molar volume. It is postulated that solute molecules are encircled by water molecules, maintaining relatively large distances between them, thereby facilitating polar interactions with the water molecules. The ($\:{V}_{\phi\:}^{0}$) values of the studied solutions increase with rising concentrations of PILs and temperature, indicating that the interactions become more pronounced at higher temperatures and elevated concentrations of PILs.

The ($\:{S}_{v}$) value serves as a criterion for evaluating solute-solute interactions, with positive ($\:{S}_{v}$) values indicating the presence of interactions between *DL-*alanine molecules. The temperature dependence of the ($\:{V}_{\phi\:}^{0}$)​has been modeled using a second-degree polynomial Eq. (3) [28]:

3 $$\:{V}_{\phi\:}^{0}=A+BT+C{T}^{2}$$

The apparent molar expansibility in infinite dilution at studied pressure was determined by applying the empirical parameters *A*, *B*, and *C*, as described by the Eq. (4) [29].

4 $$\:{E}_{\phi\:}^{0}={\left(\frac{\partial\:{V}_{\phi\:}^{0}}{\partial\:T}\right)}_{p}=B+2CT$$

The data presented in Table 3 indicate a declining trend in ($\:{V}_{\phi\:}^{0}$) values by rising the temperature. This behavior implies that as temperature rises, the water molecules are progressively released from the hydration layer. The volumetric expansion of amino acid is notably more pronounced in the presence of mono (2-hydroxyethyl) ammonium acetate compared to other protic ionic liquids (PILs). To provide a deeper understanding of this phenomenon, the apparent isobaric thermal expansion coefficient was calculated using the Eq. (5) [30].

5 $$\:\alpha\:=\frac{{E}_{\phi\:}^{0}}{{V}_{\phi\:}^{0}}$$

The calculated values of (α) for the amino acid are shown in Table 4:

**Table 4:** Parameters of the Masson equation and standard deviation of *DL-*alanine in aqeuous PILs for the apparent molar volume at different temperatures. The infinite Dilution apparent molar expansibility $\:{E}_{\phi\:}^{0}$ (cm^3^·mol^− 1^·k), isobaric thermal expansion coefficient (K^− 1^) and helper constant values at different temperatures

System	T	$\:{\varvec{V}}_{\varvec{\phi\:}}^{0}$	S_V_	E	$\:\varvec{\alpha\:}$	Hc*1000	σV_φ_
*DL-*alanine in aqueous solutions of [2-HEA]Ac 0.0501 (mol·kg^-1^)	288.15	60.874(0.021)	-2.152(0.111)	0.067	11.019		0.021
	298.15	61.303(0.016)	-1.325(0.083)	0.057	9.349	-9.769*10^− 4^	0.016
	308.15	62.134(0.029)	-1.009(0.154)	0.048	7.651		0.029
	318.15	62.345(0.098)	-1.560(0.052)	0.038	6.058		0.009
*DL-*alanine in aqueous solutions of [2-HEA]Ac 0.1001 (mol·kg^-1^)	288.15	60.388(0.032)	-0.869(0.170)	0.034	22.687		0.032
	298.15	61.094(0.015)	0.389(0.079)	0.045	17.316	-1.371*10^− 3^	0.015
	308.15	61.901(0.050)	-1.579(0.262)	0.063	12.347		0.049
	318.15	62.383(0.020)	-0.517(0.107)	0.083	7.448		0.020
*DL-*alanine in aqueous solutions of [2-HEA]Ac 0.1501 (mol·kg^-1^)	288.15	61.668(0.040)	-2.945(0.212)	0.051	8.205		0.040
	298.15	61.970(0.018)	-3.202(0.094)	0.045	7.182	-6.093*10^− 4^	0.018
	308.15	62.645(0.032)	-3.386(0.171)	0.038	6.132		0.032
	318.15	62.825(0.022)	-3.784(0.114)	0.032	5.144		0.021
*DL-*alanine in aqueous solutions of [BHEA]Ac 0.0498 (mol·kg^-1^)	288.15	60.089(0.015)	0.102(0.078)	0.159	26.428		0.015
	298.15	60.807(0.015)	0.601(0.080)	0.081	13.066	-7.805*10^− 3^	0.015
	308.15	61.444(0.017)	-0.677(0.088)	0.027	1.244		0.017
	318.15	61.601(0.016)	-0.616(0.085)	0.015	-12.231		0.016
*DL-*alanine in aqueous solutions of [BHEA]Ac 0.0999 (mol·kg^-1^)	288.15	60.007(0.032)	0.448(0.170)	0.084	13.916		0.032
	298.15	60.843(0.015)	-0.971(0.079)	0.069	11.34	-1.451*10^− 3^	0.015
	308.15	61.350(0.049)	0.285(0.262)	0.054	8.881		0.049
	318.15	61.896(0.020)	-0.109(0.107)	0.040	6.458		0.020
*DL-*alanine in aqueous solutions of [BHEA]Ac 0.1498 (mol·kg^-1^)	288.15	60.121(0.040)	1.171(0.212)	0.113	18.803		0.040
	298.15	61.073(0.018)	0.358(0.094)	0.073	11.895	-4.04*10^− 3^	0.018
	308.15	61.562(0.032)	-1.020(0.171)	0.032	5.238		0.032
	318.15	61.706(0.021)	0.918(0.114)	0.013	-1.322		0.021
*DL-*alanine in aqueous solutions of [THEA]Ac 0.0501 (mol·kg^-1^)	288.15	59.755(0.056)	0.662(0.296)	0.094	15.788		0.056
	298.15	60.105(0.035)	1.935(0.187)	0.092	15.262	-2.644*10^− 4^	0.035
	308.15	61.910(0.054)	-2.835(0.285)	0.089	14.390		0.054
	318.15	62.187(0.022)	0.919(0.114)	0.086	13.900		0.022
*DL-*alanine in aqueous solutions of [THEA]Ac 0.0998 (mol·kg^-1^)	288.15	60.876(0.032)	-2.082(0.170)	0.093	14.932		0.032
	298.15	61.204(0.015)	-0.169(0.079)	0.068	11.026	2.535*10^− 3^	0.015
	308.15	61.716(0.049)	0.324(0.262)	0.043	6.9770		0.049
	318.15	62.551(0.020)	-0.799(0.107)	0.017	2.8500		0.020
*DL-*alanine in aqueous solutions of [THEA]Ac 0.1502 (mol·kg^-1^)	288.15	61.272(0.040)	0.521(0.212)	0.062	10.168		0.040
	298.15	61.905(0.018)	0.156(0.094)	0.044	7.049	-1.866*10^− 3^	0.018
	308.15	62.093(0.032)	1.142(0.171)	0.025	4.023		0.032
	318.15	62.353(0.022)	0.205(0.114)	0.012	1.013		0.022

The standard uncertainties for molality, temperature and pressure were *u* (*m*) *=* 0.001 mol·kg^-1^, *u* (*T*) *=* 0.2 K, *u* (*P*) *=* 10.5 hPa, respectively with level of confidence 0.68

The data reveal that (α) decreases with rising temperature, a trend attributed to the disruption of Hydrogen bonds at elevated temperatures. The structural behavior of the solute (*DL-*alanine) in studied solutions containing water and protic ionic liquids (PILs) can be assessed using the Helper’s constant, which is determined through the Eq. (6) [31]:

6 $$\:{\left(\frac{\partial\:{C}_{P}}{\partial\:P}\right)}_{T}=-T{\left(\frac{{\partial\:}^{2}{V}_{\phi\:}^{0}}{\partial\:{T}^{2}}\right)}_{P}=-2CT$$

The Helper’s constant values for *DL-*alanine in (water + PILs) solutions, as presented in Table 4, provide insights into its role as a structure former or structure destructor within the solution. The positive values of the constant indicate that *DL-*alanine behaves as a structure-forming agent, facilitating the organization of water molecules. The findings indicate that hydrogen bonding interactions between *DL-*alanine and water are modulated by the coexistence of the studied PILs, resulting in a reorganization of water molecules around both *DL-*alanine and the PILs.

### Ultrasonic and compressibility properties 

The isentropic compressibility was determined using the Newton-Laplace equation, as represented by Eq. (7). Additionally, the apparent molar isentropic compressibility of ternary solution (*DL-*alanine + PILs + water) solutions was calculated applying the corresponding Eq. (8):

7 $$\:{\kappa\:}_{s}=\frac{1}{d{u}^{2}}$$

8 $$\:{\kappa\:}_{\phi\:}=\frac{{\kappa\:}_{S}{d}_{0}-d{\kappa\:}_{{S}_{0}}}{md{d}_{0}}+\frac{{\kappa\:}_{S}M}{d}$$

Here, *(u)*,* (*$\:{\kappa\:}{{s}_{0}}$_*)*,_ ($\:{\kappa\:}_{s}$*)* represent the speed of sound, the isentropic compressibility of the solvent, and the isentropic compressibility of the solution, respectively. The isentropic compressibility ($\:{K}_{s}$) and apparent molar isentropic compressibility ($\:{\kappa\:}_{\phi\:}$) data obtained in this study are summarized in Table 5:

**Table 5:** Speed of sound *U* (m·s^− 1^), isentropic compressibility κ_S_ (pa-^1^) and apparent molar isentropic compressibility $K\phi$ (m^3^·mol^− 1^·pa^− 1^) of ternary solutions in temperature range (298.15 to 318.15) K and concentration range (0.05 to 0.15) mol·kg^− 1^

m		U / m s^− 1^				κ_S_/pa-^1^				m^3^·mol^− 1^·pa^− 1^			
		288.15 K	298.15 K	308.15 K	318.15 K	288.15 K	298.15 K	308.15 K	318.15 K	288.15 K	298.15 K	308.15 K	318.15 K
*DL*-alanine 0.0501 +[2HEA]Ac													
0.0000		1471.70	1501.33	1523.71	1539.64	4.61	4.44	4.32	4.24	-3.14	-2.12	-1.83	-1.82
0.0495		1475.29	1504.47	1526.71	1542.52	4.58	4.42	4.30	4.22	-3.04	-2.26	-1.97	-1.75
0.0999		1478.86	1507.79	1529.76	1545.43	4.55	4.39	4.28	4.20	-2.99	-2.33	-1.96	-1.74
0.1493		1482.29	1510.92	1532.78	1548.28	4.52	4.30	4.25	4.18	-2.94	-2.30	-1.97	-1.73
0.1994		1485.66	1514.16	1535.76	1550.85	4.50	4.34	4.23	4.16	-2.89	-2.31	-1.95	-1.64
0.2492		1489.26	1517.49	1538.74	1553.78	4.47	4.32	4.21	4.14	-2.92	-2.34	-1.95	-1.67
0.2993		1492.34	1520.61	1541.53	1556.58	4.44	4.29	4.19	4.12	-2.82	-2.31	-1.90	-1.67
*DL*-alanine 0.0999 +[2HEA]Ac													
0.0000		1476.64	1505.84	1527.83	1543.52	4.57	4.41	4.29	4.22	-3.24	-2.15	-2.47	-2.01
0.0499		1480.20	1509.07	1531.01	1546.48	4.54	4.38	4.27	4.19	-2.95	-2.32	-2.13	-1.78
0.0996		1483.58	1512.30	1533.97	1549.30	4.52	4.36	4.25	4.17	-2.83	-2.32	-2.01	-1.72
0.1497		1487.03	1515.65	1537.05	1552.19	4.49	4.33	4.22	4.15	-2.81	-2.35	-2.01	-1.71
0.1995		1490.46	1518.70	1539.92	1554.86	4.46	4.31	4.20	4.13	-2.79	-2.28	-1.95	-1.65
0.2494		1493.82	1521.81	1542.91	1557.73	4.44	4.29	4.18	4.11	-2.75	-2.2	-1.93	-1.66
0.3005		1497.34	1525.05	1546.05	1560.66	4.41	4.26	4.16	4.09	-2.74	-2.24	-1.94	-1.67
*DL*-alanine 0.1501 +[2HEA]Ac													
0.0000		1481.70	1510.37	1532.02	1547.43	4.53	4.38	4.26	4.19	-3.06	-1.92	-1.95	-1.68
0.0496		1485.19	1513.51	1535.03	1550.35	4.51	4.35	4.24	4.17	-2.75	-2.14	-1.86	-1.68
0.1000		1488.56	1516.84	1538.01	1553.18	4.48	4.33	4.22	4.15	-2.65	-2.23	-1.82	-1.61
0.1499		1491.96	1519.96	1541.03	1556.15	4.45	4.30	4.20	4.13	-2.65	-2.20	-1.84	-1.66
0.1999		1495.36	1523.29	1543.96	1559.01	4.43	4.28	4.10	4.10	-2.65	-2.24	-1.84	-1.66
0.2493		1498.58	1526.26	1547.22	1561.46	4.40	4.26	4.15	4.09	-2.61	-2.19	-1.91	-1.58
0.2988		1502.08	1529.51	1549.92	1564.31	4.38	4.23	4.13	4.07	-2.65	-2.22	-1.86	-1.60
*DL*-alanine 0.0501+[BHEA]Ac													
0.0000		1472.64	1502.14	1524.42	1540.25	4.60	4.43	4.32	4.24	-2.72	-2.25	-2.09	-1.61
0.0500		1476.01	1505.29	1527.45	1543.05	4.57	4.41	4.29	4.22	-2.78	-2.29	-2.02	-1.70
0.1004		1479.46	1508.46	1530.39	1545.84	4.55	4.38	4.27	4.20	-2.79	-2.26	-1.94	-1.66
0.1494		1482.88	1511.74	1533.45	1548.77	4.52	4.36	4.25	4.17	-2.78	-2.30	-1.96	-1.71
0.1998		1486.23	1514.86	1536.51	1551.58	4.49	4.34	4.23	4.15	-2.77	-2.28	-1.91	-1.71
0.2489		1489.75	1518.19	1539.37	1554.33	4.46	4.31	4.20	4.13	-2.78	-2.30	-1.90	-1.67
0.3000		1493.03	1521.23	1542.28	1557.08	4.44	4.29	4.18	4.11	-2.75	-2.26	-1.90	-1.66
*DL*-alanine 0.0501+[BHEA]Ac													
0.0000		1478.03	1506.6	1529.07	1544.47	4.56	4.40	4.28	4.21	-3.63	-3.14	-1.62	-1.64
0.0504		1481.83	1510.29	1532.08	1547.29	4.53	4.37	4.26	4.19	-3.19	-2.84	-1.91	-1.62
0.0999		1485.35	1513.81	1535.03	1550.07	4.50	4.35	4.24	4.10	-3.05	-2.77	-1.90	-1.62
0.1495		1488.78	1517.02	1538.12	1552.89	4.47	4.32	4.21	4.14	-2.95	-2.61	-1.94	-1.63
0.1993		1491.78	1520.23	1541.09	1555.34	4.45	4.30	4.19	4.13	-2.76	-2.53	-1.93	-1.54
0.2496		1495.02	1523.55	1543.67	1558.12	4.42	4.27	4.17	4.11	-2.70	-2.49	-1.82	-1.54
0.2994		1498.28	1526.59	1546.55	1561.01	4.40	4.25	4.15	4.09	-2.66	-2.41	-1.81	-1.57
*DL*-alanine 0.1501+[BHEA]Ac													
0.0000		1484.24	1512.52	1533.86	1549.24	4.51	4.36	4.25	4.18	-2.42	-1.99	-1.82	-1.63
0.0499		1487.54	1515.66	1536.87	1552.03	4.49	4.33	4.23	4.15	-2.55	-2.16	-1.90	-1.58
0.1007		1490.98	1518.93	1539.86	1554.73	4.46	4.31	4.20	4.13	-2.59	-2.19	-1.86	-1.50
0.1501		1494.35	1522.15	1542.91	1557.62	4.43	4.29	4.18	4.11	-2.60	-2.21	-1.89	-1.57
0.2004		1497.81	1525.25	1546.00	1560.44	4.41	4.26	4.16	4.09	-2.61	-2.17	-1.90	-1.57
0.2497		1500.97	1528.39	1548.73	1562.95	4.38	4.24	4.14	4.07	-2.56	-2.16	-1.84	-1.50
0.2995		1504.60	1531.60	1551.67	1565.83	4.36	4.22	4.12	4.05	-2.61	-2.16	-1.82	-1.53
*DL*-alanine 0.0501+[THEA]Ac													
0.0000		1475.33	1504.63	1526.71	1542.60	4.58	4.41	4.303	4.22	-3.40	-2.44	-2.42	-1.79
0.0497		1478.89	1507.85	1529.91	1545.49	4.55	4.39	4.39	4.20	-3.00	-2.38	-2.17	-1.70
0.0979		1482.15	1510.95	1532.73	1548.16	4.53	4.36	4.36	4.18	-2.86	-2.34	-2.00	-1.61
0.1498		1485.67	1514.23	1535.89	1551.29	4.50	4.34	4.34	4.16	-2.80	-2.29	-1.97	-1.66
0.1994		1489.11	1517.56	1539.19	1554.27	4.47	4.32	4.32	4.14	-2.77	-2.31	-2.04	-1.67
0.2501		1492.51	1520.57	1541.94	1556.82	4.44	4.29	4.29	4.12	-2.73	-2.23	-1.93	-1.57
0.3003		1495.87	1523.84	1544.82	1560.09	4.42	4.27	4.27	4.09	-2.708	-2.232	-1.873	-1.638
*DL*-alanine 0.1001+[THEA]Ac													
0.0000		1483.82	1512.29	1533.75	1549.12	4.51	4.36	4.25	4.17	-2.96	-2.26	-2.25	-1.93
0.0499		1487.39	1515.61	1536.91	1552.05	4.49	4.33	4.22	4.15	-2.85	-2.35	-2.04	-1.69
0.0996		1490.72	1518.98	1539.95	1554.83	4.46	4.31	4.20	4.13	-2.70	-2.38	-1.98	-1.62
0.1500		1494.25	1522.26	1542.99	1557.66	4.43	4.28	4.18	4.11	-2.71	-2.34	-1.94	-1.60
0.1993		1497.68	1525.42	1545.96	1560.60	4.41	4.26	4.16	4.09	-2.70	-2.30	-1.92	-1.64
0.2492		1501.04	1529.01	1548.82	1563.17	4.38	4.23	4.14	4.07	-2.66	-2.36	-1.87	-1.58
0.2994		1504.05	1531.89	1551.94	1566.29	4.36	4.21	4.11	4.05	-2.55	-2.26	-1.89	-1.63
*DL*-alanine 0.1501+[THEA]Ac													
0.0000		1492.41	1520.06	1540.83	1555.69	4.45	4.30	4.20	4.13	-2.96	-2.70	-2.16	-1.78
0.0499		1496.02	1523.38	1543.95	1558.55	4.42	4.28	4.17	4.11	-2.75	-2.23	-1.90	-1.56
0.0999		1499.36	1526.53	1546.91	1561.28	4.40	4.25	4.15	4.09	-2.59	-2.14	-1.82	-1.50
0.1499		1502.73	1529.61	1549.96	1564.13	4.37	4.23	4.13	4.00	-2.56	-2.09	-1.82	-1.52
0.1948		1505.71	1532.51	1552.62	1566.46	4.35	4.21	4.11	4.05	-2.53	-2.10	-1.80	-1.47
0.2498		1509.47	1536.05	1555.89	1569.62	4.32	4.18	4.09	4.03	-2.53	-2.12	-1.80	-1.50
0.2997		1511.98	1539.37	1558.94	1572.40	4.30	4.16	4.07	4.01	-2.37	-2.15	-1.81	-1.50

The standard uncertainties for molality, temperature and pressure were *u* (*m*) = 0.002 mol·kg^− 1^, *u* (*T*) = 0.02 K, *u* (*P*) = 10 hPa, respectively with level of confidence 0.68. The combined standard uncertainty for speed of sound was, *u* (*u*) = 0.6 m·s^− 1^ with level of confidence 0.68

**Fig. 3:**
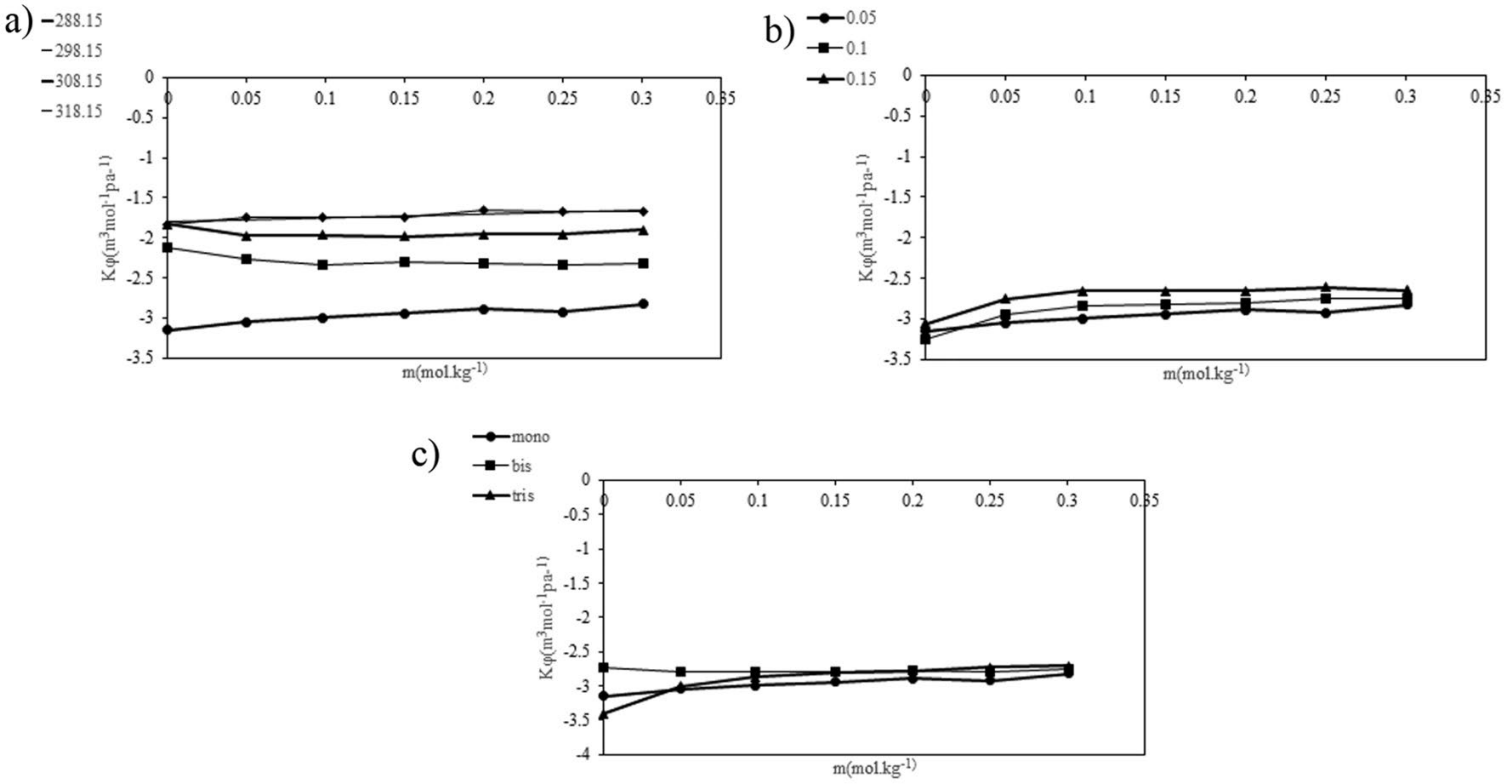
Variation of apparent molar isentropic compressibility ( $K\phi$) with, **a**) temperature, **a**) concentration of PILs and **c**) cation size of the PILs

Analysis of ($\:{K}_{s}$) reveals an inverse relationship with the concentration of *DL-*alanine. The variation of ($\:{\kappa\:}_{\phi\:}$) with *DL-*alanine molality, the concentration of the PILs, and temperature is shown in Fig. 3:

These findings indicate that the ($\:{K}_{s}$) values decrease with increasing the concentration of *DL-*alanine. This effect can be attributed to the disruption of the three-dimensional hydrogen bond network among water molecules, which weakens the cohesive forces within the liquid and reduces its overall resistance to flow. The standard partial molar isentropic compressibility of *DL-*alanine was calculated using the specified Eq. (9) [32];

9 $$\:{\kappa\:}_{\phi\:}={\kappa\:}_{\phi\:}^{0}+{S}_{k}m$$

where, ($\:{\kappa\:}_{\phi\:}^{0}$) is the partial molar isentropic compressibility and ($\:{S}_{k}$) symbol is the empirical parameters of the Eq. (9). The parameters obtained for the studied solutions are presented in Table 6:

**Table 6:** The values of S_K_ (m^3^·mol^− 3/2^·kg^1/2^·pa^− 1^), B_K_ (m^3^mol^− 2^.kg·pa^− 1^), $\:{\kappa\:}_{\phi\:}^{0}$ (m^3^·mol^− 1^·pa^− 1^) obtained for each mixture and standard deviation at the experimental temperatures from Eq. (7)

System	T	$\:{\varvec{\kappa\:}}_{\varvec{\phi\:}}^{0}$	S_K_	σ$\:{\varvec{\kappa\:}}_{\varvec{\phi\:}}$
*DL-*alanine in aqueous solutions of [2-HEA]Ac 0.0501 (mol·kg^-1^)	288.15	-3.146	0.382	0.025
	298.15	-2.121	-0.920	0.023
	308.15	-1.833	-0.904	0.017
	318.15	-1.825	0.293	0.025
*DL-*alanine in aqueous solutions of [2-HEA]Ac 0.1001 (mol·kg^-1^)	288.15	-3.244	1.653	0.029
	298.15	-2.156	-1.153	0.026
	308.15	-2.470	1.932	0.033
	318.15	-2.012	1.297	0.023
*DL-*alanine in aqueous solutions of [2-HEA]Ac 0.1501 (mol·kg^-1^)	288.15	-3.060	1.816	0.033
	298.15	-1.927	-1.382	0.032
	308.15	-1.958	0.687	0.029
	318.15	-1.684	0.013	0.029
*DL-*alanine in aqueous solutions of [BHEA]Ac 0.0499 (mol·kg^-1^)	288.15	-2.722	-0.444	0.011
	298.15	-2.250	-0.236	0.016
	308.15	-2.091	0.395	0.027
	318.15	-1.614	-0.512	0.021
*DL-*alanine in aqueous solutions of [BHEA]Ac 0.0999 (mol·kg^-1^)	288.15	-3.630	1.922	0.042
	298.15	-3.148	1.260	0.031
	308.15	-1.620	-1.925	0.039
	318.15	-1.648	-1.450	0.030
*DL-*alanine in aqueous solutions of [BHEA]Ac 0.1498 (mol·kg^-1^)	288.15	-2.423	-0.795	0.022
	298.15	-1.999	-1.045	0.021
	308.15	-1.824	-0.535	0.025
	318.15	-1.633	0.354	0.031
*DL-*alanine in aqueous solutions of [THEA]Ac 0.0502 (mol·kg^-1^)	288.15	-3.400	2.216	0.035
	298.15	-2.445	0.211	0.021
	308.15	-2.426	1.423	0.054
	318.15	-1.798	0.609	0.040
*DL-*alanine in aqueous solutions of [THEA]Ac 0.0998 (mol·kg^-1^)	288.15	-2.966	0.578	0.039
	298.15	-2.267	-0.710	0.033
	308.15	-2.253	1.122	0.021
	318.15	-1.937	1.502	0.033
*DL-*alanine in aqueous solutions of [THEA]Ac 0.1502 (mol·kg^-1^)	288.15	-2.969	1.094	0.046
	298.15	-2.709	2.878	0.044
	308.15	-2.163	1.573	0.026
	318.15	-1.783	1.296	0.025

The combined standard uncertainty for apparent molar isentropic compressibility were, 10 ^14^ u ($\:{\kappa\:}_{\phi\:}^{0}$) = 0.7 m^3^·mol^− 1^·pa^− 1^

A negative value for this parameter indicates that the amino acid, as the solute, is surrounded by (PILs + water) molecules, which resist compression more effectively compared to the bulk solvent. Furthermore, the ($\:{\kappa\:}_{\phi\:}^{0}$) values decrease with increasing temperature, attributed to the intrinsic thermal expansion at elevated temperatures, where pressure-induced volume expansion becomes more pronounced.

### Viscosity results

Viscosity is a measure of a fluid’s resistance to flow, influenced by intermolecular interactions and temperature [33]. In solutions, viscosity can vary significantly based on solute concentration and the nature of the solute and solvent. For instance, adding a solute like sugar to water increases viscosity due to enhanced molecular interactions. Understanding viscosity is crucial in fields like pharmaceuticals and food science, where it affects mixing, stability, and flow behavior. The dynamic and kinematic viscosities of *DL-*alanine in aqueous solutions containing PILs, measured over the temperature range of (288.15 to 318.15) K, are summarized in Table 7.

**Table 7:** Dynamic viscosity and kinematic viscosity of *DL-*alanine in PILs aqueous solutions at different temperatures

*m*	*η*_k_				*η*_D_			
mol·kg^− 1^	mm^2^·S^− 1^				mPa·s			
	**288.15 K**	**298.15 K**	**308.15 K**	**318.15 K**	**288.15 K**	**298.15 K**	**308.15 K**	**318.15 K**
*DL-*alanine in aqueous solutions of [2-HEA]Ac 0.0501 (mol·kg^-1^)								
0.0000	1.1553	0.9050	0.7392	0.6184	1.156	0.903	0.736	0.618
0.0495	1.1664	0.9187	0.7442	0.6251	1.169	0.916	0.742	0.625
0.0999	1.1791	0.9252	0.7520	0.6301	1.183	0.926	0.751	0.631
0.1493	1.1926	0.9357	0.7622	0.6372	1.199	0.938	0.762	0.636
0.1994	1.2061	0.9462	0.7685	0.6441	1.214	0.950	0.769	0.643
0.2492	1.2258	0.9558	0.7760	0.6526	1.231	0.961	0.778	0.650
0.2993	1.2349	0.9671	0.7841	0.6596	1.246	0.973	0.787	0.656
*DL-*alanine in aqueous solutions of [2-HEA]Ac 0.1001 (mol·kg^-1^)								
0.0000	1.1703	0.9190	0.7672	0.6425	1.173	0.918	0.766	0.639
0.0499	1.1876	0.9293	0.7726	0.6458	1.189	0.930	0.771	0.643
0.0996	1.2084	0.9442	0.7783	0.6498	1.207	0.946	0.779	0.647
0.1497	1.2228	0.9593	0.7841	0.6557	1.231	0.962	0.785	0.653
0.1995	1.2382	0.9711	0.7918	0.6597	1.250	0.974	0.791	0.659
0.2494	1.2501	0.9801	0.7965	0.6634	1.262	0.986	0.801	0.663
0.3005	1.2664	0.9915	0.8038	0.6686	1.284	0.998	0.805	0.668
*DL-*alanine in aqueous solutions of [2-HEA]Ac 0.1501 (mol·kg^-1^)								
0.0000	1.1979	0.9401	0.7647	0.6478	1.202	0.941	0.762	0.643
0.0496	1.2116	0.9496	0.7706	0.6526	1.217	0.952	0.770	0.650
0.1003	1.2220	0.9622	0.7809	0.6584	1.230	0.966	0.782	0.655
0.1499	1.2372	0.9705	0.7889	0.6633	1.247	0.975	0.790	0.662
0.1999	1.2541	0.9840	0.8005	0.6695	1.268	0.987	0.801	0.669
0.2493	1.2658	0.9909	0.8087	0.6769	1.279	0.998	0.813	0.678
0.2988	1.2806	1.0019	0.8179	0.6818	1.296	1.011	0.821	0.685
*DL-*alanine in aqueous solutions of [BHEA]Ac 0.0499 (mol·kg^-1^)								
0.0000	1.1682	0.9176	0.7459	0.6282	1.168	0.916	0.743	0.621
0.0502	1.1781	0.9256	0.7526	0.6327	1.181	0.926	0.751	0.626
0.1004	1.1923	0.9348	0.7604	0.6418	1.196	0.936	0.759	0.635
0.1494	1.2112	0.9453	0.7706	0.6479	1.218	0.948	0.771	0.644
0.1998	1.2252	0.9582	0.7803	0.6541	1.232	0.962	0.782	0.654
0.2489	1.2348	0.9711	0.7894	0.6626	1.245	0.976	0.792	0.663
0.3001	1.2502	0.9790	0.7947	0.6678	1.262	0.986	0.798	0.668
*DL-*alanine in aqueous solutions of [BHEA]Ac 0.0999 (mol·kg^-1^)								
0.0000	1.1829	0.9293	0.7551	0.6302	1.186	0.931	0.753	0.627
0.0504	1.1946	0.9374	0.7612	0.6365	1.201	0.939	0.762	0.634
0.0999	1.2151	0.9539	0.7715	0.6451	1.222	0.958	0.775	0.641
0.1495	1.2266	0.9619	0.7808	0.6520	1.236	0.966	0.782	0.652
0.1993	1.2427	0.9743	0.7911	0.6587	1.253	0.981	0.794	0.659
0.2496	1.2582	0.9849	0.7975	0.6640	1.271	0.992	0.801	0.665
0.2994	1.2802	0.9936	0.8053	0.6708	1.295	1.002	0.812	0.672
*DL-*alanine in aqueous solutions of [BHEA]Ac 0.1498 (mol·kg^-1^)								
0.0000	1.2269	0.9605	0.7784	0.6519	1.229	0.962	0.776	0.651
0.0499	1.2476	0.9748	0.7915	0.6591	1.251	0.977	0.787	0.658
0.1007	1.2582	0.9836	0.7967	0.6691	1.265	0.988	0.802	0.668
0.1501	1.2768	1.0012	0.8103	0.6766	1.287	1.002	0.812	0.677
0.2004	1.2951	1.0125	0.8195	0.6838	1.304	1.018	0.822	0.685
0.2497	1.3049	1.0208	0.8272	0.6892	1.320	1.030	0.832	0.692
0.2995	1.3214	1.0334	0.8372	0.6973	1.339	1.044	0.843	0.702
*DL-*alanine in aqueous solutions of [THEA]Ac 0.0501 (mol·kg^-1^)								
0.0000	1.1907	0.9357	0.7603	0.6371	1.194	0.937	0.758	0.638
0.0497	1.2018	0.9426	0.7650	0.6415	1.206	0.944	0.764	0.643
0.0979	1.2131	0.9506	0.7718	0.6468	1.220	0.953	0.771	0.649
0.1498	1.2269	0.9614	0.7797	0.6517	1.235	0.965	0.781	0.653
0.1994	1.2397	0.9726	0.7886	0.6586	1.251	0.978	0.789	0.659
0.2501	1.2574	0.9801	0.7952	0.6628	1.269	0.986	0.796	0.664
0.3003	1.2669	0.9926	0.8022	0.6685	1.281	1.001	0.804	0.671
*DL-*alanine in aqueous solutions of [THEA]Ac 0.0998 (mol·kg^-1^)								
0.0000	1.2317	0.9560	0.7801	0.6583	1.241	0.958	0.780	0.657
0.0499	1.2405	0.9692	0.7862	0.6621	1.251	0.973	0.789	0.661
0.0996	1.2539	0.9777	0.7943	0.6671	1.268	0.983	0.798	0.668
0.1501	1.2688	0.9905	0.8052	0.6734	1.281	0.997	0.809	0.673
0.1993	1.2816	1.0047	0.8136	0.6781	1.296	1.009	0.818	0.680
0.2492	1.2944	1.0126	0.8224	0.6818	1.310	1.022	0.827	0.685
0.2994	1.3105	1.0235	0.8285	0.6881	1.328	1.034	0.834	0.692
*DL-*alanine in aqueous solutions of [THEA]Ac 0.1502 (mol·kg^-1^)								
0.0000	1.2686	0.9906	0.8088	0.6757	1.279	0.996	0.811	0.679
0.0499	1.2834	1.0036	0.8145	0.6819	1.296	1.010	0.819	0.685
0.0999	1.2952	1.0133	0.8231	0.6858	1.309	1.021	0.828	0.691
0.1499	1.3076	1.0224	0.8287	0.6921	1.327	1.032	0.836	0.696
0.1948	1.3194	1.0313	0.8365	0.6968	1.341	1.042	0.843	0.701
0.2498	1.3389	1.0437	0.8439	0.7017	1.361	1.056	0.852	0.707
0.2997	1.3505	1.0536	0.8506	0.7068	1.378	1.062	0.860	0.713

^a^ The standard uncertainties associated with molality, temperature, and pressure were determined as u (m) = 0.001 mol·kg^− 1^, u (T) = 0.2 K and u (P) = 10.5 hPa, respectively, with a confidence level of 0.68. The standard combined uncertainty for viscosity was approximately u (η) = 0.02 mPa·s corresponding to a confidence level of 0.68

The data indicate that both dynamic and kinematic viscosities increase with higher concentrations of *DL-*alanine and PILs.

This trend indicates that as the molality of *DL*-*alanine* and PILs increases, the frequency of molecular collisions per unit volume rises, which enhances intermolecular interactions, such as hydrogen bonding and ionic interactions. These stronger interactions restrict molecular mobility, leading to an increase in viscosity. Conversely, all studied systems demonstrate a decrease in viscosity with increasing temperature, highlighting reduced resistance to flow at elevated temperatures. This behavior can be attributed to the increased kinetic energy of molecules, which weakens hydrophilic-ionic and hydrophilic-hydrophobic interactions, allowing for greater molecular movement. The dynamic viscosity data were further analyzed using the Jones-Dole equation [34]:

10 $$\:{\eta\:}_{r}=\frac{\eta\:}{{\eta\:}_{0}}=1+A\sqrt{c}+Bc$$

In this equation, (*η*) represents the viscosity of the solution, while ($\:{\eta\:}_{0}$) refers to the viscosity of the solvent. The parameter *A* is associated with long-range Coulombic interactions between solute molecules, known as the Falkenhagen coefficient. For non-electrolyte solutes, the values of the *A* coefficient are typically negligible. Additionally, $\:{S}_{v}$another parameter used to evaluate solute-solute interactions, exhibits a similar trend to the *A* coefficient and is therefore often disregarded.

The viscosity *B*-coefficient is an empirical parameter used to evaluate solute-solvent interactions. This parameter is influenced by the size, shape, and structural effects arising from solute-solvent interactions. High positive values of the *B*-coefficient indicate robust solute-solvent interactions, corroborating the findings of V_ϕ_^0^ which also serves as an indicator of solute-solvent interactions.

As shown in Table 8, the *B*-coefficient for all systems exhibits a decreasing trend with increasing temperature, indicating a reduction in solute-solvent interactions as the temperature rises.

**Table 8 The viscosity:** *B*-coefficient (dm^3^·mol^− 1^) of *DL*-alanine in aqueous solution of PILs obtained from Jones-Dole equation.

*T*	*B*
K	dm^3^·mol^− 1^
*DL-*alanine in aqueous solutions of [2-HEA]Ac 0.0501 (mol·kg^-1^)	
288.15	0.259(0.01)
298.15	0.243(0.05)
308.15	0.223(0.03)
318.15	0.216(0.04)
*DL-*alanine in aqueous solutions of [2-HEA]Ac 0.1001 (mol·kg^-1^)	
288.15	0.387(0.02)
298.15	0.361(0.04)
308.15	0.357(0.08)
318.15	0.321(0.04)
*DL-*alanine in aqueous solutions of [2-HEA]Ac 0.1501 (mol·kg^-1^)	
288.15	0.453(0.09)
298.15	0.430(0.02)
308.15	0.422(0.05)
318.15	0.415(0.01)
*DL-*alanine in aqueous solutions of [BHEA]Ac 0.0499 (mol·kg^-1^)	
288.15	0.320(0.02)
298.15	0.305(0.07)
308.15	0.293(0.05)
318.15	0.290(0.01)
*DL-*alanine in aqueous solutions of [BHEA]Ac 0.0999 (mol·kg^-1^)	
288.15	0.421(0.02)
298.15	0.390(0.01)
308.15	0.367(0.03)
318.15	0.325(0.01)
*DL-*alanine in aqueous solutions of [BHEA]Ac 0.1498 (mol·kg^-1^)	
288.15	0.607(0.02)
298.15	0.579(0.07)
308.15	0.548(0.04)
318.15	0.524(0.09)
*DL-*alanine in aqueous solutions of [THEA]Ac 0.0499 (mol·kg^-1^)	
288.15	0.400(0.08)
298.15	0.377(0.05)
308.15	0.337(0.01)
318.15	0.330(0.02)
*DL-*alanine in aqueous solutions of [THEA]Ac 0.0998( mol·kg^-1^)	
288.15	0.407(0.01)
298.15	0.421(0.07)
308.15	0.403(0.05)
318.15	0.378(0.02)
*DL-*alanine in aqueous solutions of [THEA]Ac 0.1502 (mol·kg^-1^)	
288.15	0.589(0.01)
298.15	0.582(0.03)
308.15	0.562(0.01)
318.15	0.536(0.08)

The standard uncertainties associated with molality, temperature, and pressure were u (m) = 0.001 mol·kg^− 1^, u (T) = 0.2 K and u (P) = 10.5 hPa, respectively, corresponding to a confidence level of 0.68. The standard combined uncertainty for viscosity was approximately u (η) = 0.02 mPa·s with a confidence level of 0.68

Additionally, the viscosity *B*-coefficient values are consistently higher than the *A*-coefficient values, suggesting that interactions between the solute and solvent are more influential than solute-solute interactions.

### Refractive index and molar refraction

The refractive indices of ternary solutions containing *DL-*alanine, protic ionic liquids (PILs), and water were measured over a temperature range of (288.15 to 318.15) K. The experimentally determined refractive index values for these solutions are presented in Table 9.

**Table 9:** Refractive index and the molar refraction of the ternary aqueous solutions of *DL-*alanine in the presence of PILs at different temperatures

*m*	*n*_D_				*R*_M_			
mol·kg^− 1^	288.15 K	298.15 K	308.15 K	318.15 K	288.15 K	298.15 K	308.15 K	318.15 K
*DL*-alanine 0.0501 + [2-HEA]Ac								
0.0000	1.3347	1.3338	1.3326	1.3312	3.7228	3.7215	3.7189	3.7134
0.0497	1.3352	1.3348	1.3336	1.3319	3.7335	3.7396	3.7371	3.7285
0.0993	1.3358	1.3356	1.3344	1.3329	3.7475	3.7558	3.7534	3.7469
0.1496	1.3364	1.3366	1.3351	1.3339	3.7613	3.7739	3.7684	3.7651
0.2001	1.3373	1.3372	1.3358	1.3348	3.7782	3.7880	3.7837	3.7824
0.2499	1.3383	1.3379	1.3367	1.3355	3.7930	3.8030	3.8008	3.7976
0.2992	1.3389	1.3386	1.3375	1.3364	3.8100	3.8181	3.8171	3.8148
*DL*-alanine 0.1001 + [2-HEA]Ac								
0.0000	1.3356	1.3345	1.3335	1.3321	3.7249	3.7239	3.7234	3.7182
0.0494	1.3364	1.3353	1.3343	1.3327	3.7408	3.7401	3.7396	3.7321
0.0997	1.3371	1.3361	1.3352	1.3334	3.7558	3.7551	3.7547	3.7473
0.1498	1.3378	1.3367	1.3357	1.3342	3.7707	3.7702	3.7699	3.7636
0.1993	1.3389	1.3377	1.3365	1.3349	3.7897	3.7883	3.7860	3.7787
0.2496	1.3397	1.3385	1.3374	1.3359	3.8058	3.8045	3.8033	3.7970
0.2994	1.3404	1.3391	1.3382	1.3367	3.8211	3.8188	3.8176	3.8134
*DL*-alanine 0.1501 + [2-HEA]Ac								
0.0000	1.3363	1.3352	1.3342	1.3331	3.7272	3.7263	3.7258	3.7234
0.0501	1.3370	1.3360	1.3349	1.3341	3.7422	3.7424	3.7421	3.7407
0.1005	1.3379	1.3368	1.3357	1.3348	3.7594	3.7587	3.7575	3.7572
0.1501	1.3387	1.3374	1.3365	1.3354	3.7754	3.7728	3.7737	3.7714
0.1993	1.3395	1.3382	1.3375	1.3362	3.7914	3.7889	3.7919	3.7877
0.2500	1.3402	1.3391	1.3384	1.3371	3.8063	3.8049	3.8090	3.8038
0.3002	1.3412	1.3399	1.3393	1.3377	3.8242	3.8218	3.8262	3.8188
*DL*-alanine 0.0501 + [BHEA]Ac								
0.0000	1.3349	1.3337	1.3324	1.3315	3.7204	3.7191	3.7155	3.7150
0.0500	1.3359	1.3347	1.3334	1.3323	3.7391	3.7371	3.7336	3.7311
0.1004	1.3366	1.3356	1.3342	1.3329	3.7540	3.7539	3.7498	3.7454
0.1494	1.3372	1.3365	1.3351	1.3336	3.7717	3.7711	3.7660	3.7605
0.1998	1.3381	1.3372	1.3358	1.3342	3.7875	3.7861	3.7821	3.7746
0.2489	1.3391	1.3383	1.3366	1.3352	3.8059	3.8054	3.7983	3.7909
0.3000	1.3400	1.3390	1.3374	1.3357	3.8222	3.8205	3.8145	3.8061
*DL*-alanine 0.1001 + [BHEA]Ac								
0.0000	1.3356	1.3344	1.3329	1.3320	3.7217	3.7198	3.7144	3.7139
0.0498	1.3364	1.3351	1.3339	1.3328	3.7377	3.7349	3.7326	3.7302
0.1001	1.3373	1.3358	1.3346	1.3336	3.7546	3.7498	3.7477	3.7463
0.1496	1.3382	1.3368	1.3352	1.3345	3.7715	3.7679	3.7617	3.7613
0.1998	1.3389	1.3377	1.3361	1.3352	3.7865	3.7850	3.7789	3.7787
0.2494	1.3397	1.3387	1.3368	1.3359	3.8025	3.8024	3.7941	3.7940
0.2994	1.3405	1.3394	1.3378	1.3367	3.8185	3.8183	3.8124	3.8102
*DL*-alanine 0.1501 + [BHEA]Ac								
0.0000	1.3368	1.3353	1.3343	1.3333	3.7278	3.7230	3.7227	3.7213
0.0498	1.3378	1.3362	1.3352	1.3341	3.7457	3.7401	3.7399	3.7376
0.1004	1.3387	1.3371	1.3362	1.3351	3.7629	3.7564	3.7562	3.7560
0.1501	1.3395	1.3382	1.3369	1.3359	3.7788	3.7745	3.7733	3.7722
0.1997	1.3403	1.3387	1.3376	1.3367	3.7949	3.7897	3.7885	3.7881
0.2495	1.3410	1.3394	1.3383	1.3374	3.8098	3.8047	3.8036	3.8025
0.2996	1.3418	1.3403	1.3392	1.3381	3.8258	3.8219	3.8209	3.8185
*DL*-alanine 0.0498 + [THEA]Ac								
0.0000	1.3354	1.3342	1.3331	1.3322	3.7218	3.7198	3.7184	3.7179
0.0488	1.3362	1.3350	1.3340	1.3330	3.7377	3.7358	3.7355	3.7342
0.0987	1.3370	1.3358	1.3347	1.3337	3.7533	3.7516	3.7504	3.7493
0.1497	1.3378	1.3367	1.3355	1.3345	3.7697	3.7691	3.7670	3.7661
0.1994	1.3385	1.3374	1.3363	1.3353	3.7847	3.7842	3.7832	3.7825
0.2472	1.3393	1.3381	1.3369	1.3359	3.8010	3.7996	3.7977	3.7971
0.3001	1.3401	1.3389	1.3378	1.3368	3.8171	3.8159	3.8153	3.8148
*DL*-alanine 0.0998 + [THEA]Ac								
0.0000	1.3372	1.3361	1.3350	1.3340	3.7294	3.7286	3.7273	3.7261
0.0500	1.3380	1.3369	1.3358	1.3347	3.7454	3.7447	3.7436	3.7413
0.1001	1.3390	1.3378	1.3366	1.3354	3.7633	3.7618	3.7597	3.7566
0.1499	1.3395	1.3383	1.3372	1.3360	3.7764	3.7750	3.7740	3.7709
0.1999	1.3403	1.3391	1.3379	1.3369	3.7924	3.7901	3.7891	3.7881
0.2491	1.3411	1.3397	1.3386	1.3377	3.8085	3.8052	3.8044	3.8043
0.2993	1.3417	1.3406	1.3395	1.3385	3.8228	3.8224	3.8215	3.8205
*DL*-alanine 0.1501 + [THEA]Ac								
0.0000	1.3389	1.3378	1.3367	1.3357	3.7360	3.7355	3.7344	3.7332
0.0498	1.3397	1.3386	1.3375	1.3364	3.7521	3.7517	3.7507	3.7485
0.0997	1.3405	1.3393	1.3382	1.3372	3.7682	3.7669	3.7659	3.7648
0.1494	1.3413	1.3401	1.3389	1.3378	3.7843	3.7831	3.7812	3.7791
0.1999	1.3419	1.3407	1.3396	1.3386	3.7975	3.7964	3.7956	3.7945
0.2500	1.3429	1.3417	1.3405	1.3393	3.8163	3.8153	3.8136	3.8106
0.3002	1.3438	1.3425	1.3412	1.3402	3.8331	3.8313	3.8287	3.8278

Standard uncertainties (*u*) for each variable are *u* (*m*) = 0.0002 mol·kg^− 1^, *u* (*T*) = 0.01 K; *u* (*n*_D_) = 0.001; *u* (P) = 1.05 hPa

The temperature dependence of the refractive index follows a trend similar to that of solution density, as outlined in Table 3. According to the data in Table 9, the refractive index decreases with increasing temperature and increases with higher concentrations of *DL-*alanine.

To calculate the molar refraction, the Lorentz-Lorenz equation was applied [35]:

11 $$\:{R}_{M}=\frac{{n}_{D}^{2}-1}{{n}_{D}^{2}+2}\cdot\:\frac{{x}_{1}\cdot\:{M}_{1}+{x}_{2}\cdot\:{M}_{2}}{d}$$

**Fig. 4:**
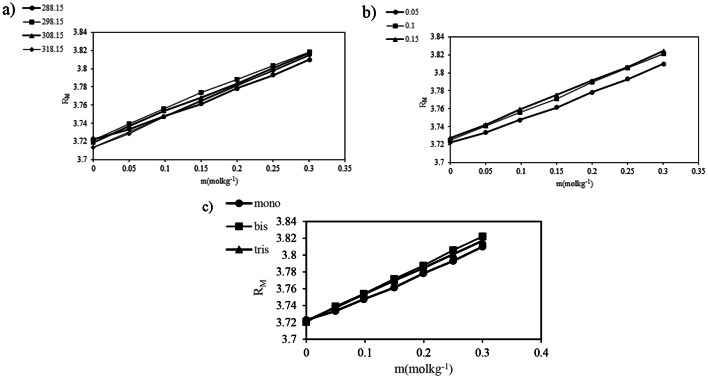
molar refraction variation of ternary solutions$(RM)$ with, **a**) temperature, **b**) concentration of PILs and **c**) cation size of the PILs

In this equation *(*$\:{x}_{1}$*) is* mole fraction *and (M*_1_), (*M*_2_)​are molecular weights of each component in the mixture, while (*d)* denotes the density of the solution. The values of (*n*_D_)​ and molar refraction are provided in Table 9. Molar refraction is an important parameter that reflects solute-solvent interactions and the molecular polarizability within the solution. As shown in Table 9, the molar refraction values increase with higher concentrations of PILs, suggesting enhanced polarizability in the solutions studied and indicating strong interactions between PILs and *DL-*alanine. Increased molar refraction indicates a substance’s ability to polarize in response to an electric field. This is linked to enhanced polarizability, which refers to the ease with which electron clouds can be distorted. As more electrons are present, or as the molecular structure allows for greater distortion, both molar refraction and polarizability increase. Thus, a direct relationship exists where higher polarizability leads to greater molar refraction. Furthermore, the variations in molar refraction with temperature, PIL concentration, and the cation size of PILs are illustrated in Fig. 4:

Based on Fig. 4 As the size of cations in protic ionic liquids increases, the molar refraction also tends to increase. Larger cations have more electrons and a more substantial electron cloud, which enhances their polarizability. This increased polarizability leads to a greater ability to distort in an electric field, contributing to higher molar refraction. Consequently, the relationship reflects the interplay between cation size and electron distribution.

### Hydration behavior interpretation

This study demonstrates that *DL-*alanine has the smallest cavity volume among the molecules analyzed, indicating its more compact molecular structure. This compactness suggests that the hydration behavior of *DL-*alanine may differ from that of the other solutes examined. The smaller cavity volume and surface area imply fewer potential sites for hydrogen bonding (H-bonding) with solvent molecules. Also, *DL-*alanine exhibits the least negative dielectric energy (hydration energy) among the studied molecules, indicating weaker solute-solvent interactions. The relatively less negative hydration energy further suggests that the hydrogen bonding between *DL-*alanine and the solvent is weaker compared to the other solutes.

In contrast, the mono-, bis-, and tris (2-hydroxyethyl) ammonium acetate molecules exhibit progressively larger cavity volumes and surface areas, signifying a greater spatial requirement and more complex interactions with the solvent than *DL-*alanine. Their more negative dielectric energies indicate that these molecules engage in stronger and more favorable interactions with the solvent. The increased cavity volumes and surface areas provide more opportunities for hydrogen bond (H-bond) formation with the solvent, enhancing stronger and more favorable H-bonding interactions.

Additionally, the Helper’s constant is introduced as a useful parameter to evaluate the structure-making or structure-breaking behavior of *DL-*alanine in the presence of the examined PILs. Positive values of this constant suggest that *DL-*alanine exhibits structure-making behavior, facilitating the rearrangement of the hydrogen bonding network between the PILs and water molecules. This implies that the presence of the studied PILs may influence the hydrogen bonding interactions between *DL-*alanine and water molecules, potentially altering the hydration dynamics.

In conclusion, the findings highlight the distinct hydration behavior of *DL-*alanine compared to the studied PILs. The reduced surface area, smaller cavity volume, and less negative hydration energy of *DL-*alanine indicate its compact nature and weaker hydrogen bonding interactions with the solvent. In contrast, the protic ionic liquids (PILs), with their larger cavity volumes, expanded surface areas, more negative hydration energies, and higher capacity for hydrogen bonding, demonstrate more intricate interactions with the solvent. The temperature-dependent behavior and the impact of the PILs emphasize the significant role that hydrogen bonding plays in governing the hydration dynamics of *DL-*alanine.

## Conclusions

This investigation reveals that *DL-*alanine interacts more weakly with water molecules compared to the surrounding protic ionic liquids (PILs), such as mono-, bis-, and tris-(2-hydroxyethyl) ammonium acetate. The weaker interactions observed for *DL-*alanine are attributed to its compact molecular structure and lower negative dielectric energy. In contrast, the PILs, with their larger molecular size and more complex structures, engage more strongly with water molecules, primarily through hydrogen bonding. Temperature variations significantly affect the hydration layer around *DL-*alanine, leading to a greater release of water molecules compared to the PIL solutions. The positive value of the Helper constant indicates that *DL-*alanine facilitates the ordering of water molecules in its surrounding environment. In contrast, the PILs may alter the hydrogen bonding between *DL-*alanine and water by reorganizing the water molecules and forming their own hydrogen bonds with the solvent.

## Supplementary Information

Below is the link to the electronic supplementary material.

Supplementary Material 1

## Data Availability

The authors confirm that the data supporting the findings of this study are available within the manuscript, figures, tables, and supporting information files.
