# Supplementary material for: Influence of 2-hydroxyethylammonium acetate-based protic ionic liquids on the thermophysical properties of aqueous DL-alanine solutions
Source: BMC Chem. 2025 Aug 13;19(1):240. doi: 10.1186/s13065-025-01603-1 (PMC12351930; doi:10.1186/s13065-025-01603-1)
Supplement: Supplementary file 1 — Supplementary Material 1 [file 13065_2025_1603_MOESM1_ESM.docx]

**“Supporting Information”**

Influence of 2-Hydroxyethylammonium Acetate-Based Protic Ionic Liquids on the Thermophysical Properties of Aqueous *DL*-Alanine Solutions

Mohammad Amin Morsali, Hemayat Shekaari*

* *Department of Physical Chemistry, Faculty of Chemistry, University of Tabriz, Tabriz, Iran*

Corresponding author Email address: [hemayatt@yahoo.com](mailto:hemayatt@yahoo.com)

Tel.: +98-41-33393139. Fax: +98-41-33340191.


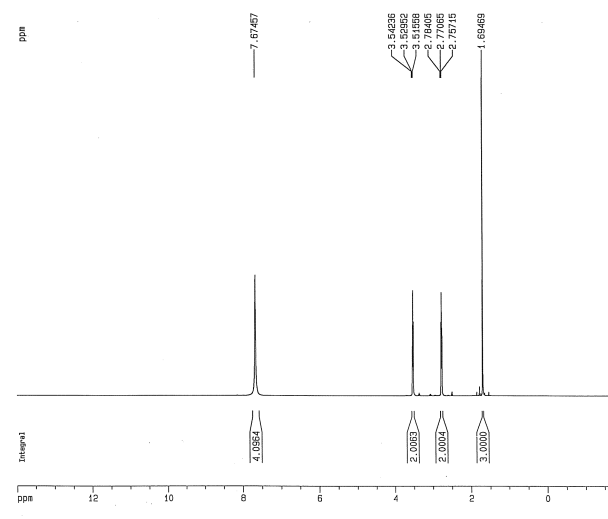


**Figure S1.** ^1^HNMR spectrum of the synthesized PIL [2-HEA]Ac.


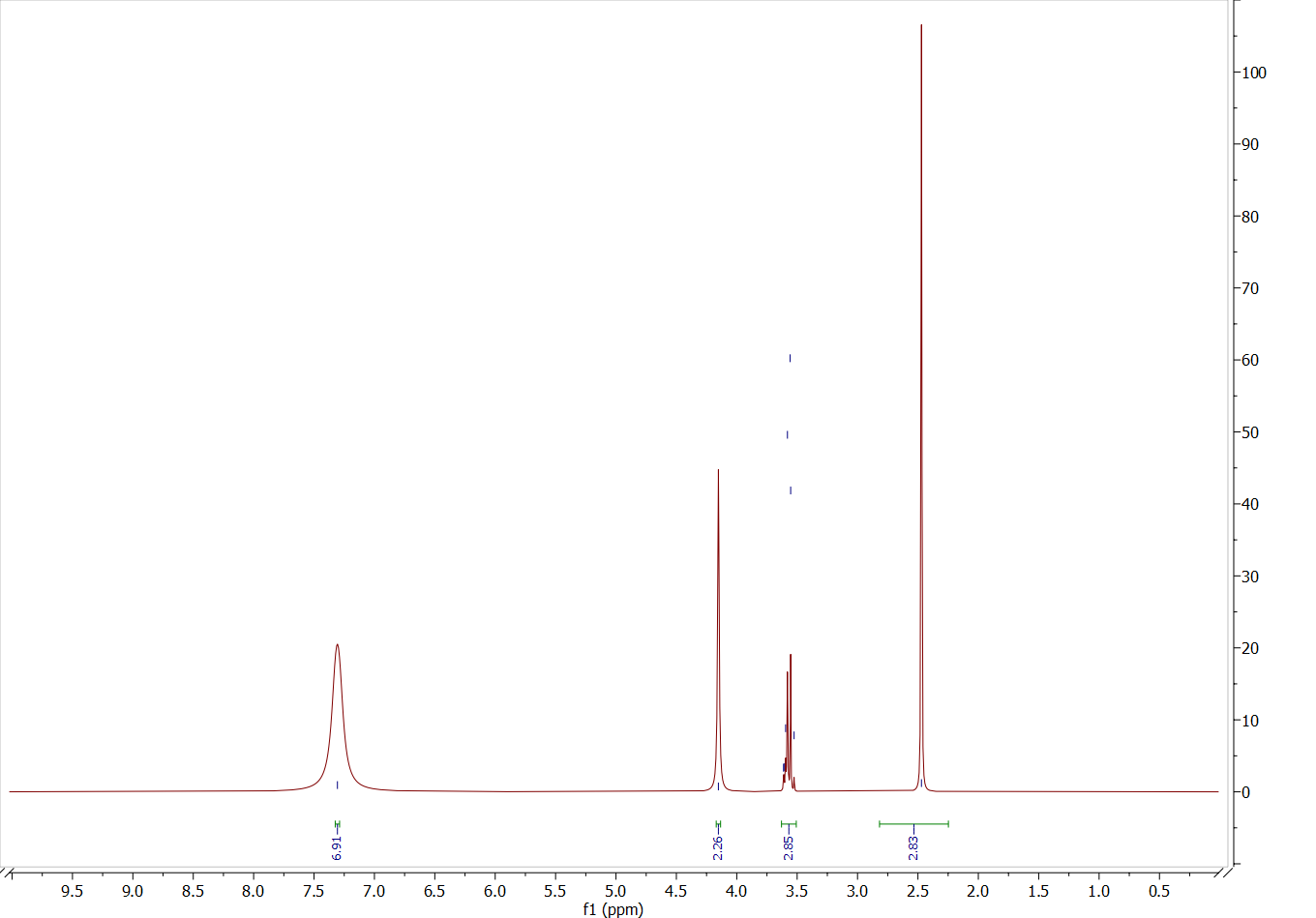


**Figure S2.** ^1^HNMR spectrum of the synthesized PIL [bis-2-HEA]Ac

**
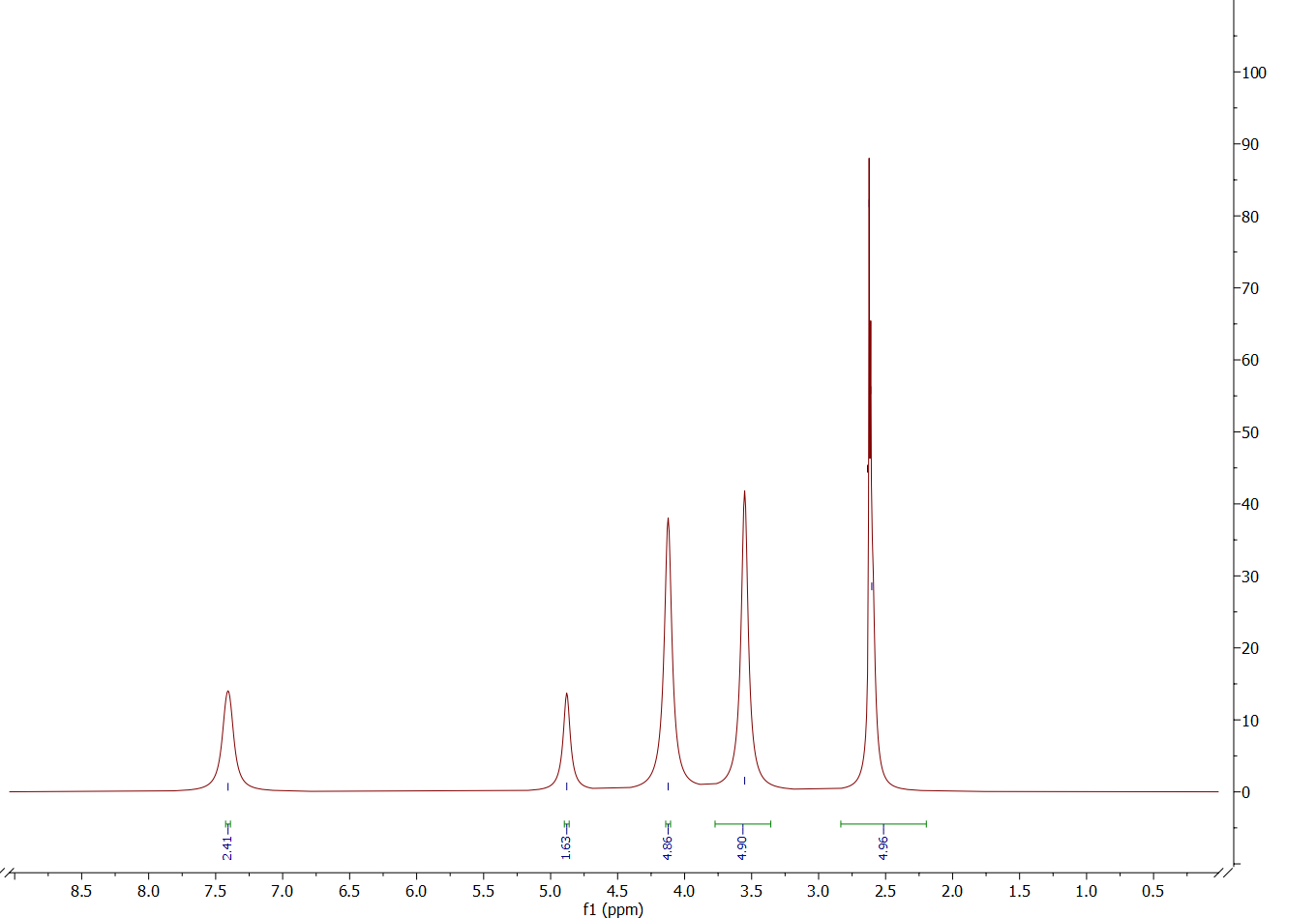
**

**Figure S3.** ^1^HNMR spectrum of the synthesized PIL [tris-2-HEA]Ac

**Figure S4.** *FT-IR spectrum of the [2-HEA]Ac*

**Figure S5**. FT-IR spectrum of the [bis-2-HEA]Ac.

**Figure S6**. FT-IR spectrum of the [tris-2-HEA]Ac.
